# Habitat differentiation and conservation gap of *Magnolia biondii*, *M. denudata*, and *M. sprengeri* in China

**DOI:** 10.7717/peerj.6126

**Published:** 2019-03-12

**Authors:** Chuangye Song, Huiming Liu

**Affiliations:** 1 State Key Laboratory of Vegetation and Environmental Change, Institute of Botany Chinese Academy of Sciences, Beijing, China; 2 Satellite Environment Center, Ministry of Environmental Protection, Beijing, China

**Keywords:** Traditional Chinese medicine, Maxent, Human disturbance, In situ conservation, Ex situ conservation

## Abstract

The flower buds of *Magnolia biondii*, *M. denudata*, and *M. sprengeri* are the materials of Xinyi, a traditional Chinese medicine. The harvest of flower buds and habitat fragmentation caused by human disturbance heavily threatens the natural regeneration and survival of these three *Magnolia* species. With the aim to support the conservation and improve the effectiveness of conservation, we performed an assessment on habitat suitability, influences of environmental variables on habitat suitability, and the conservation gap of these three *Magnolia* species, based on the Maxent modeling method. The results indicated that: (1) altitude, annual mean temperature, extreme temperature, temperature fluctuation, annual precipitation, and extreme precipitation are the most influential environmental variables for the distribution of *M. sprengeri*, *M. biondii*, and *M. denudata*; (2) obvious habitat differentiations were observed among *M. biondii*, *M. denudata*, and *M. sprengeri. M. sprengeri* tends to be located in further northern areas with higher altitudes, lower temperatures, and lower precipitation compared to *M. biondii* and *M. denudata*; and (3) a large proportion of suitable habitats have been left without protection. Woodland and forest shared the largest area out of the suitable habitats. However, grassland, agricultural land, residential land, and mining and industry areas also occupied large areas of suitable habitats.

## Introduction

Xinyi is a famous traditional Chinese medicine. It is made from the dried flower buds of *Magnolia biondii* Pampan., *M. denudata* Desr., and *M. sprengeri* Pampan. (Chinese Pharmacopoeia Commission, 2015). Xinyi was first recorded in the ancient book, “Sheng Nong’s Herbal Classic”, in which it was regarded as a high-grade medicine. Xinyi was known to be used as a medicinal material in the treatment of cold-headaches, nasal obstruction, and nasosinusitis (Wang, 2006; Huang & Yan, 2009; Ma, Zhang & Chen, 2017). In recent years, Xinyi has become more widely used in medicine, and its demand has greatly increased. However, overharvesting of flower buds exerts negative influences on the natural regeneration of these three *Magnolia* species (Jiang & Sheng, 1997). In addition to this, habitat fragmentation and cutting for timber heavily threatened the survival of *Magnolia* taxa (Jiang & Sheng, 1997; Wang & Jiang, 2001; Cires et al., 2013). *M. biondii*, *M. denudata*, and *M. sprengeri* were listed as vulnerable species in the China Species Red List (Wang & Xie, 2004; Rivers et al., 2016). Therefore, it is necessary to strengthen the conservation of wild resources of these *Magnolia* species.

Understanding the relationship between the distribution of plants and environmental factors (such as altitude, slope, temperature, precipitation, soil, and geology) is the crucial step for us to develop effective strategies for the conservation of endangered or threatened species (Bell, 2001; Zhang & Ma, 2008). However, few studies have been performed to analyze the relationship between the distribution of these three *Magnolia* species and environmental factors.

Habitat conservation is an effective strategy for the conservation of endangered plants (Maxted et al., 2008). In order to protect *M. biondii*, *M. denudata*, and *M. sprengeri* effectively, it is necessary to find the most suitable habitats for them, and then perform in situ or ex situ conservation. Presently, in the context of the “Returning Farmland to Forests” program in China, *M. biondii*, *M. denudata*, and *M. sprengeri* have been widely planted in many regions to advance the development of local economies. The artificial cultivation and propagation of these species has been promoted by local governments. The quality of Xinyi is closely related to environmental factors. Improper selection of cultivation sites will not only lead to the waste of land resources and financial loss to farmers, but also to the diminishment of medicine quality (Shi et al., 2015). Therefore, assessment of habitat suitability is required in order to support the selection of cultivation sites for these species.

Various methods have been used to estimate the habitat suitability by using presence-only data. This includes BIOCLIM (Busby, 1986), DOMAIN (Carpenter, Gillison & Winter, 1993), GARP (Stockwell, 1999), NFA (Hirzel et al., 2002), and Maxent (Phillips, Anderson & Schapire, 2006). Among these modeling methods, Maxent has a superior performance on the simulation of habitat and species distribution compared to that of other models (Elith et al., 2006; Wisz et al., 2008; Phillips, Anderson & Schapire, 2006; Phillips et al., 2009). It was widely used in the prediction of species distribution and habitat suitability assessment (Harte et al., 2008; Yackulic et al., 2013).

Nature reserves provide refuges for many threatened or endangered species. Many nature reserves were established to conserve biodiversity and habitats in China. According to published data, the areas of national reserves and other nature reserves are 960,000 and 504,800 km^2^, respectively (Wang et al., 2016). Therefore, in this research, we have focused on the effects of nature reserves on the conservation of *Magnolia* species. In addition to this, human disturbances have a great influence on the conservation of biodiversity. Therefore, in this research, we aim to determine the status of human disturbance within the area of suitable habitats.

In conclusion, the aims of this research were to (1) explore the relationship between environmental variables and habitat suitability, (2) project the potential distribution of suitable habitats in China, and (3) assess the conservation gap and the status of human disturbance within the suitable habitats. We hope that this research can provide technical support for the conservation and cultivation of *M. biondii*, *M. denudata*, and *M. sprengeri*.

## Data and methods

### Species distribution data

**Figure 1:**
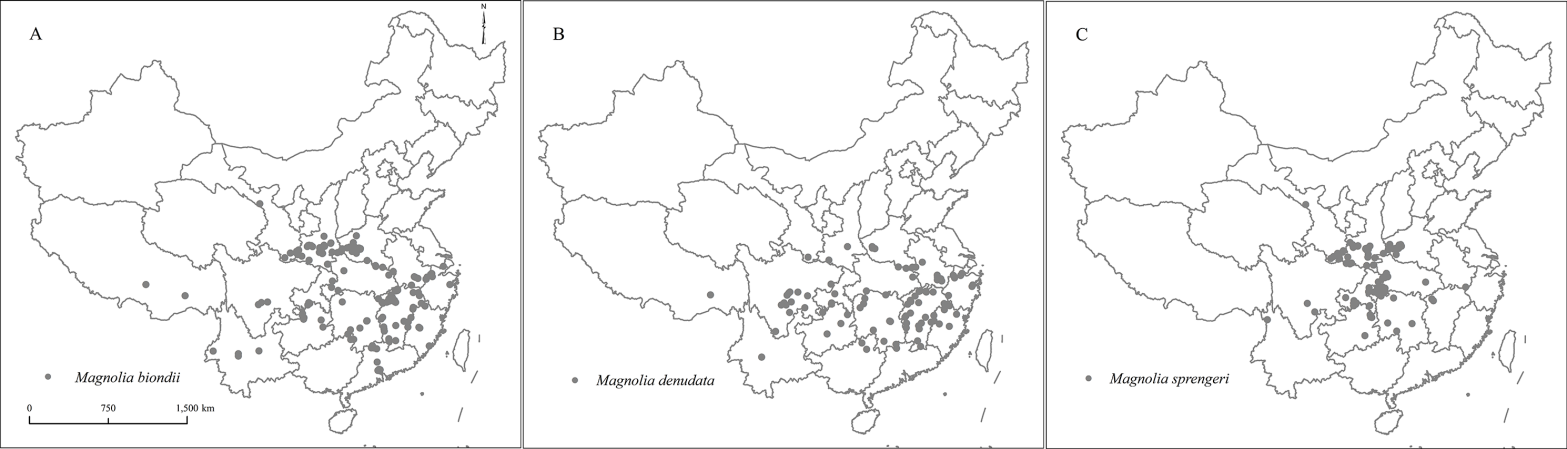
The location where the specimens of *Magnolia biondii*, *Magnolia denudata,* and *Magnolia sprengeri* were collected. A–C are, respectively, the location map of *Magnolia biondii*, *Magnolia denudata*, and *Magnolia sprengeri*.

The location information of *M. biondii*, *M. denudata*, and *M. sprengeri* was derived from specimens preserved in the herbarium (http://www.cvh.ac.cn/) (Fig. 1). In the past, longitudes and latitudes were not recorded because of the lack of global positioning systems (GPS). However, the addresses where the specimens were collected were recorded. Those place names were used to infer longitude and latitude using Google Earth (https://www.google.com/earth/).

Duplicate records were deleted from the dataset. In addition to this, records of specimens made from cultivated trees were excluded from this dataset. Finally, 162, 93, and 90 specimen records were used for the modeling of *M. biondii*, *M. denudata*, and *M. sprengeri*, respectively.

### Environmental variables

Nineteen bioclimatic variables were downloaded from the WorldClim website (http://www.worldclim.org/) (Table 1). They represented the averages for the years 1970–2000, with a spatial resolution of 2.5′ (Hijmans et al., 2005).

**Table 1:** Environmental variables used for model fitting.

	Environmental variables
Topography	Altitude
	Slope
	Aspect
Temperature	Annual mean temperature
	Mean diurnal range (mean of monthly (max temp − min temp))
	Isothermality (bio2/bio7) (*100)
	Temperature seasonality (standard deviation *100)
	Max temperature of warmest month
	Min temperature of coldest month
	Temperature annual range (bio5–bio6)
	Mean temperature of wettest quarter
	Mean temperature of driest quarter
	Mean temperature of warmest quarter
	Mean temperature of coldest quarter
Precipitation	Annual precipitation
	Precipitation of wettest month
	Precipitation of driest month
	Precipitation seasonality (coefficient of variation)
	Precipitation of wettest quarter
	Precipitation of driest quarter
	Precipitation of warmest quarter
	Precipitation of coldest quarter

Topographic variables (altitude, slope, and aspect) were extracted from the ASTER Global Digital Elevation Model (GDEM). The ground resolution of the ASTER GDEM is 30 m, the vertical accuracy is estimated to be 20 m, and the horizontal accuracy is 30 m, with 95% confidence (Tachikawa et al., 2011). The ASTER GDEM was resampled and interpolated to the same spatial resolution as that of the bioclimatic variable (2.5′). Slope and aspect were calculated using the spatial analyst tools provided by ArcGIS (V9.3). For the aspect, the northern slope was set as 0° and increased gradually to 180° at the southern slope. This served to transform the aspect from a circular variable into a continuous variable.

In order to decrease the influences of high correlations between pairs of environmental variables on model fitting, we set 0.7 as the maximum correlation coefficient allowed between environmental variables (Dormann et al., 2013). Table S1 presents the correlation coefficients between pairs of environmental variables. Altitude, slope, aspect, mean diurnal range, temperature seasonality, minimum temperature of coldest month, mean temperature of wettest quarter, annual precipitation, precipitation seasonality, and precipitation of warmest quarter were used to perform the model fitting.

### Nature reserves and land use/cover data

Vector data for national nature reserves were provided by the Nanjing Institute of Environmental Sciences, Ministry of Environmental Protection, China (http://www.nies.org/). Land use/cover data of China (2015) with one km spatial resolution were provided by the Resources and Environment Data Center of the Chinese Academy of Sciences (http://www.resdc.cn/).

### Model fitting

Maxent software was employed to assess the habitat suitability of *M. biondii*, *M. denudata*, and *M. sprengeri* in this research. Among the records of specimens, 25% were randomly selected for the test data and the remainder was used as training data to fit the model. The output format of the model values was cloglog, and the model gives an estimate between 0 and 1 for probability of presence.

Other parameters were set as the following: “maximum iterations” was 500, “maximum number of background points” was 10,000, “replicates” was 1, and “replicated run type” was cross-validate. The “convergence threshold” was set as 0.00001, and the regularization multiplier was set as 1.

The Maxent software presented different approaches to assess the contribution of environmental variables: percent contribution, permutation importance, and jackknife test.

Percent contribution and permutation importance are based on the gain of the mode; gain is similar to deviance, in that it can be used to measure the goodness of model fitting (Phillips, 2017). Percent contribution represents the contribution that the environmental variable made to the fitted model, and depends on the path the model adopted to achieve the optimal solution (Songer et al., 2012; Smart et al., 2012).The permutation importance depends on the final model, and is denoted by the change of area under the receiver operating curve (AUC) due to the variation in the values of predictors between presence and background points (Songer et al., 2012). In addition, when high correlation exists between pairs of environmental variables, the percent contributions should be interpreted with caution (Phillips, 2017). The results of percent contribution and permutation contribution are shown in Table S2.

The jackknife test was conducted based on the training gain, test gain, and AUC. In the jackknife test, a model was first developed by using all the environmental variables. Then, each environmental variable was omitted in turn, and a model was created with the other environmental variables. Additionally, a model was developed using only one environmental variable. Through comparing the change in model gain under different model fitting strategies, the importance of each environmental variable was assessed. The result of the jackknife test is shown in Fig. S1.

Based on Table S2 and Fig. S1, we ranked these environmental variables according to the contribution of each variable (Tables S3–S5) for *M. biondii*, *M. denudata*, and *M. sprengeri*.

Response curves were also fitted to show how the habitat suitability changed when environmental variables varied from minimum to maximum. In order to diminish the influences of correlation among environmental variables, each response curve was fitted by using only one corresponding environmental variable, omitting other variables. The response curves of *M. biondii*, *M. denudata*, and *M. sprengeri* are presented in Figs. S2–S4, respectively*.* The suitable range of environmental variables of these three species (Table 2) were estimated based on the response curves of habitat suitability to environmental variables.

**Table 2:** The habitat characteristics of *Magnolia biondii*, *Magnolia denudata,* and *Magnolia sprengeri*.

	Magnolia biondii	Magnolia denudata	Magnolia sprengeri
Altitude	250–850	120–620	1,000–2,000
Annual mean temperature	13–14.5	16–18	11–13.8
Mean diurnal range	6.5–9	4–8	7.5–9.5
Isothermality	24–29	23–28	27–31
Temperature seasonality	700–850	690–830	700–800
Max temperature of warmest month	29–32.5*	33.1–33.8	22–25
Min temperature of coldest month	−5 to 1.5	−1.2 to 2.5	−7 to −1
Temperature annual range	26–31.5	26–31	27–31
Mean temperature of wettest quarter	19–23*	21.3–22.2	19–20
Mean temperature of driest quarter	1.2–8*	6.9–8.8	1–2.9
Mean temperature of warmest quarter	24.2–24.6	>30.5	19–24*
Mean temperature of coldest quarter	1.2–6.5	3.5–8.7	−1 to 4
Annual precipitation	1,400–2,100	1,375–2,125	690–1,200
Precipitation of wettest month	260–290	>240	206–219
Precipitation of driest month	>30	>32	7.5–35
Precipitation seasonality	47.5–70	44–63	57–75
Precipitation of wettest quarter	575–950	600–1,000	425–630
Precipitation of driest quarter	>125	>120	25–110
Precipitation of warmest quarter	530–625	520–600	450–650*
Precipitation of coldest quarter	>150	>140	25–125

**Note:**

* The range of environmental variable was estimated when the habitat suitability higher than 0.7.

### Model validation

In many studies, only presence data was available for species distribution modeling; no negative records (absence) could be used to measure specificity. It was hard to use the receiver operating characteristic curves (ROC) to assess the performance of the fitted models. Phillips, Anderson & Schapire (2006) presented an approach that could avoid this problem by distinguishing presence from random, rather than presence from absence. Therefore, ROCs were used to validate the fitted models in the Maxent software.

In ROCs, the *y*-axis indicated the sensitivity, and the *x*-axis indicated the false-positive fraction (1-specificity). The AUC was calculated as a measure of prediction success. The AUC ranged from 0 to 1, with a value higher than 0.5 indicating a better-than-random performance event (Fielding & Bell, 1997). The following system was often used to indicate the performance of the model (Swets, 1988): poor (0.5–0.6), fair (0.6–0.7), good (0.7–0.8), very good (0.8–0.9), and excellent (0.9–1.0). In this research, the AUC of the training data and test data were both higher than 0.9 (Fig. S5). This indicated that the performance of the fitted models was excellent.

### Threshold selection

The output ASC II grid is a continuous probability data that ranges from 0 to 1. Many thresholds were used to transform the continuous probability into binary data; those used include 0.5 (Waltari & Guralnick, 2009), 0.8 (Ramírez-Barahona et al., 2009), the minimum predicted value (Phillips, Anderson & Schapire, 2006), the 10th percentile training presence threshold (Brito et al., 2009), the 20th percentile training presence threshold (Donegan & Avendaño, 2010), the threshold that results in a sensitivity of 95% (Newbold et al., 2009), and maximization of the sum of sensitivity and specificity (Liu, White & Newell, 2013; Liu, Newell & White, 2016).

Different methods were adopted (including the above methods) to estimate thresholds by the Maxent software and the thresholds estimated in this research were all lower than 0.5 (Tables S6–S8). Li et al. (2014) deemed that grids with probabilities higher than 0.6 could be regarded as the most suitable habitats (core area). Liu, White & Newell (2013) and Liu, Newell & White (2016) investigated which thresholds can be used confidently with presence-only data, and the thresholds estimated by most methods were lower than 0.8.

The threshold should be chosen based on the objective for generating the species distribution model (Wilson et al., 2005). In this research, the main objective was to determine the location of the most suitable habitats, and provide guidance for the conservation of these *Magnolia* species. The area of the suitable habitats cannot be too large for the consideration of conservation cost. Therefore, a large threshold should be selected to transform the continuous probability into binary data.

Based on the objective of this research and the thresholds used in previous studies, we selected 0.8 as the threshold to transform the continuous probability into binary data in this research. Grids with a probability higher than 0.8 were regarded as suitable habitats.

### Assessment of conservation gap and human disturbance

The suitable habitats of *M. biondii*, *M. denudata*, and *M. sprengeri* were merged into a single suitable habitat layer by using the “merge” tool provided by ArcGIS (v9.3). The overlay analysis of nature reserves and suitable habitats was performed to assess the conservation gap of these species. Additionally, the overlayer analysis of land use and suitable habitats was conducted and the percent of each land use type within the suitable habitats was calculated to indicate the degree of human disturbance.

## Results

### The importance of environmental variables

The contribution of environmental variables were presented in Table S2 and Fig. S1. Among these 10 selected variables, altitude is the most influential topographic variable on habitat suitability (Table S3), temperature seasonality and min temperature of coldest month were regarded as the most important temperature variables (Table S4), annual precipitation was recognized as the most influential precipitation variables (Table S5).

We could also see that high correlations existed between altitude and annual mean temperature, max temperature of warmest month, mean temperature of warmest quarter, temperature seasonality and temperature annual range, min temperature of coldest month and mean temperature of driest quarter (Table S1). Additionally, high correlations existed between the annual precipitation and precipitation of the wettest month, precipitation of the driest month, precipitation of the wettest quarter, and precipitation of the driest quarter (Table S1).

Based on these, we concluded that altitude, annual mean temperature, extreme temperature, temperature fluctuation, annual precipitation, and extreme precipitation are the most influential environmental variables for the distribution of these *Magnolia* species.

### Habitat characteristics of *M. biondii*, *M. denudata*, and *M. sprengeri*

Compared to *M. biondii* and *M. denudata*, *M. sprengeri* tended to grow in higher altitude areas; they could tolerate greater temperature fluctuations (annual range), lower extreme temperatures, less precipitation, and greater precipitation fluctuations (Table 2). Between *M. biondii* and *M. denudata*, *M. denudata* favors lower and warmer areas with smaller temperature fluctuations, more precipitation and smaller precipitation fluctuations (Table 2).

### Spatial distribution of suitable habitats

**Figure 2:**
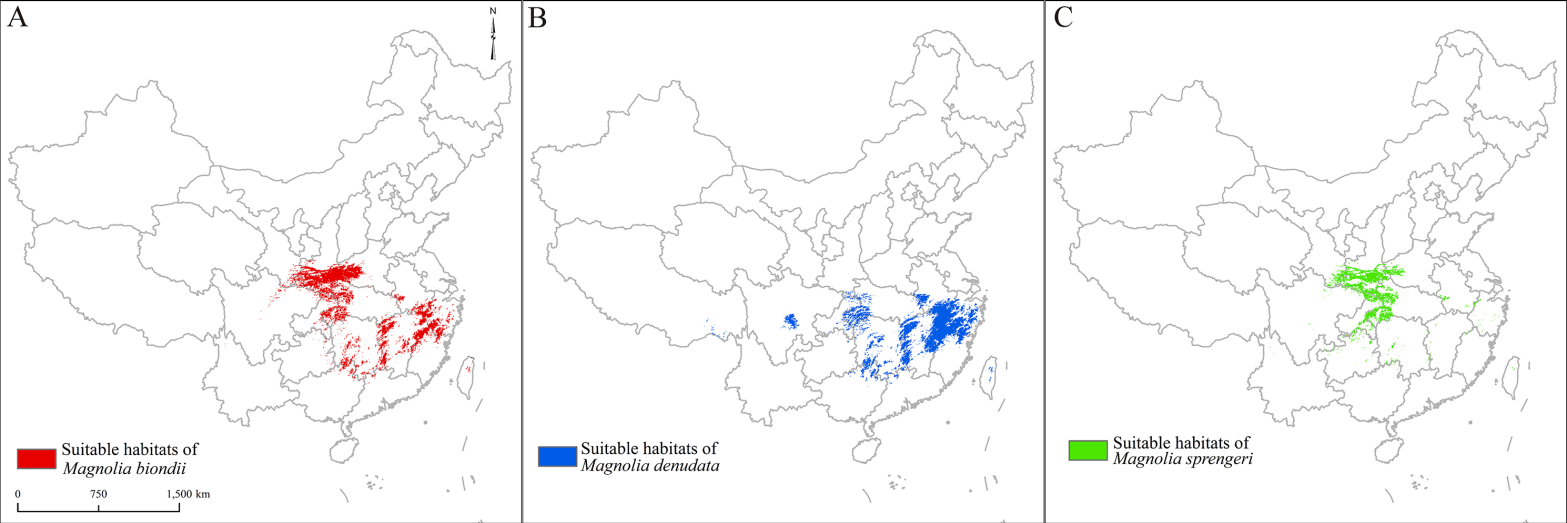
Spatial distribution of suitable habitats of *Magnolia biondii*, *Magnolia denudata,* and *Magnolia sprengeri*. A–C are, repectively, the suitable habitats of *Magnolia biondii*, *Magnolia denudata,* and *Magnolia sprengeri.*

The suitable habitats of *M. denudata* were mainly located in the middle sub-tropics (Fig. 2). Small areas of suitable habitats could also be found in the southeastern areas of Tibet and Taiwan. The spatial distribution patterns of *M. biondii* were similar to that of *M. denudata*. *M. sprengeri* extended to locations in further northern areas (Fig. 2), and the area of its suitable habitat was smaller than that of *M. denudata* and *M. biondii*.

### Conservation gap and human disturbance

The overlay analysis of nature reserves and suitable habitats indicated that only 4.75% of the suitable habitats were under the protection of national reserves (Fig. S6). Large areas of suitable habitats were left without any protection.

**Figure 3:**
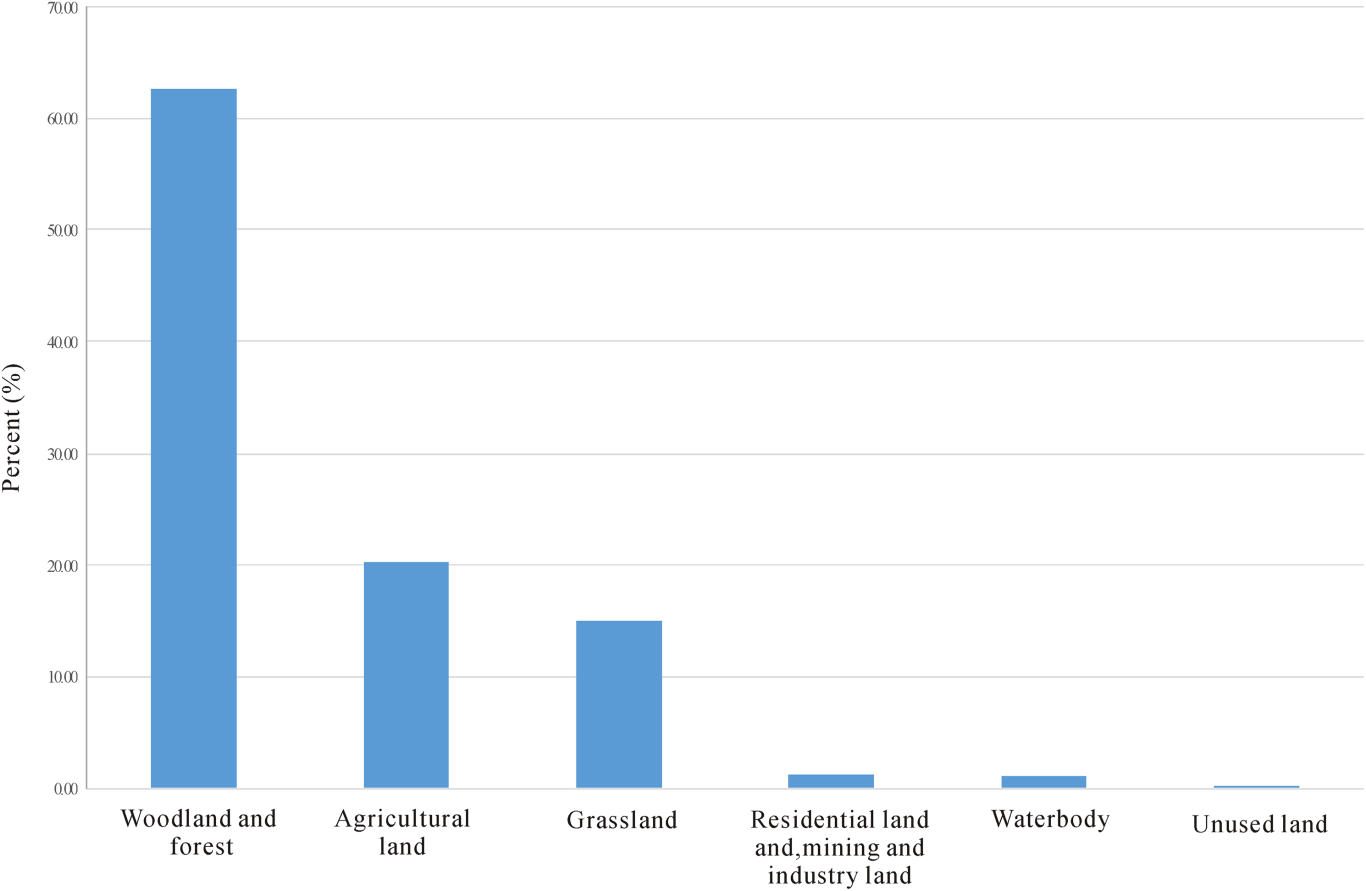
Percent of land use/cover types of the suitable habitats.

The overlay analysis of land use and suitable habitats indicated that woodland and forest shared the largest area of the suitable habitats (Fig. 3; Fig. S7). However, in the land use/cover data, orchards, mulberry fields, tea gardens, and other kinds of plantation forest were not differentiated from natural woodlands and forest. It was difficult to determine the percent of plantation forest in the woodland and forest categories. Agricultural land, residential land, mining land, and industrial areas also occupied a large portion of suitable habitats (Fig. 3). In addition to this, grassland is a very important land cover within the suitable habitats. However, in the southern area of China, most grasslands are secondary vegetation caused by deforestation. Heavy human disturbance still exists in these suitable habitats.

## Discussion

Distinct habitat differentiations were observed among *M. biondii, M. denudata*, and *M. sprengeri* (Table 2; Fig. 2). This information is very important for us to perform in situ or ex situ conservation of these *Magnolia* species. Moreover, this information can assist in the selection of suitable areas to cultivate these species. However, a deficiency of this study is that only variables of topography, temperature, and precipitation were used to estimate the habitat suitability; other variables, such as soil, hydrology, and geology, were not included in the modeling. This might cause some uncertainties in the assessment of habitat suitability (Gaston, 2003). In addition, the geographical coordinates of the specimens used in the modeling were not measured by GPS. They were inferred from the recorded names of the villages and towns located near the sites where the specimens were collected. Villages and towns are usually located in flat areas with relative low altitudes. This might lead to mismatches between the altitude derived from the Digital Elevation Model (DEM), and the actual altitude of collection.

Nature reserves are the most important and effective refuges for wild plants and wild germplasm resources. However, only a small percent of the suitable habitats are under the protection of national nature reserves (Fig. S6). Heavy human disturbance still exists in these suitable habitats (Fig. 3). This remind us that more measures and actions should be taken to conserve the wild resources of *M. biondii*, *M. denudata*, and *M. sprengeri*. However, in this research, only national nature reserves were used to estimate the conservation gap. Other nature reserves, such as provincial nature reserves, municipal nature reserves, county level nature reserves, national forest park, scenic spots, and geological parks were not included in this research. Therefore, the percent of protected suitable habitats could be higher than 4.75% (Fig. S6).

The habitat suitabilities estimated by our research provide a scientific base for the selection of priority conservation areas of *M. biondii*, *M. denudata*, and *M. sprengeri*. Besides habitat suitabilities, genetic diversity should also be considered for the conservation of endangered species populations (Cires et al., 2013). Genetic diversity was regarded as the key to the survival of a species or population (Beardmore, 1983; Antonovics, 1984; Barrett & Kohn, 1991). It is essential for us to understand the genetic variation of inter-populations and genetic diversity of intra-populations of endangered species when we develop strategies for in situ and ex situ conservation activities (Hogbin & Peakall, 1999). Previous studies have found that the genetic diversity or genetic variation in a population is not correlated with the population size (Zhang et al., 2010). Therefore, genetic diversity within different sizes of populations and genetic variation among different populations should be fully assessed. More attention should be paid to the conservation of populations with high genetic diversity and large amounts of “private” fragments to prevent further loss of genetic diversity (Zhang et al., 2010).

China is the world center of distribution for Magnoliaceae; more than 40% of the species originated from southern China (Wang & Jiang, 2001; Cicuzza, Newton & Oldfield, 2007). In past decades, Magnoliaceae species in China have suffered heavy human disturbance, such as the harvesting of timber and medicinal material, as well as deforestation (Wang & Jiang, 2001). Many taxa of Magnoliaceae are threatened due to overexploitation and habitat destruction (Cicuzza, Newton & Oldfield, 2007; Cires et al., 2013). According to the “China Biodiversity Red List,” 71.7% of Magnoliaceae species are classified as “Threatened” (Ministry of Ecology and Environment of China and Chinese Academy of Sciences, 2013). Therefore, more studies should be performed on more threatened species; field surveys of threatened species and assessments of habitat suitability and genetic diversity are required to support the ex situ and in situ conservation of Magnoliaceae species.

## Conclusions

(1) Altitude, annual mean temperature, extreme temperature, temperature fluctuation, annual precipitation, and extreme precipitation are the most influential environmental variables for the distribution of *M. sprengeri*, *M. biondii*, and *M. denudata*.

(2) Obvious habitat differentiation was found among *M. biondii*, *M. denudata*, and *M. sprengeri*. Compared to *M. biondii* and *M. denudata*, *M. sprengeri* tended to be located in further northern areas with higher altitudes, lower temperatures, and lower precipitation.

(3) Large proportions of suitable habitats were left without any protection. Heavy levels of human interference still exist in the suitable habitats of these three *Magnolia* species.

## Supplemental Information

10.7717/peerj.6126/supp-1 Supplemental Information 1 Correlation coefficients between pairs of environmental variables.

10.7717/peerj.6126/supp-2 Supplemental Information 2 The percent contribution and permutation importance of environmental variables.

10.7717/peerj.6126/supp-3 Supplemental Information 3 The rank of topographic variables based on variable contribution.

10.7717/peerj.6126/supp-4 Supplemental Information 4 The rank of temperature variables based on variable contribution.

10.7717/peerj.6126/supp-5 Supplemental Information 5 The rank of precipitation variables based on variable contribution.

10.7717/peerj.6126/supp-6 Supplemental Information 6 Thresholds estimated by Maxent for the fitted model of *Magnolia biondii*.

10.7717/peerj.6126/supp-7 Supplemental Information 7 Thresholds estimated by Maxent for the fitted model of *Magnolia denudata*.

10.7717/peerj.6126/supp-8 Supplemental Information 8 Thresholds estimated by Maxent for the fitted model of *Magnolia sprengeri*.

10.7717/peerj.6126/supp-9 Supplemental Information 9 Contribution of environmental variables based on regularized training gain,test gain and area under receiver operating characteristic curve. The x-axis is the regularized training gain which represents the contribution of environmental variable on the model fitting (The gain is closely related to deviance, a measure of goodness of fit). The red bar indicates the regularized training gain when the model was fitted by all variables. The blue bar indicates the regularized training gain when the model was fitted with only this variable. The turquoise bar indicates the regularized training gain when the model was fitted without this variable.

10.7717/peerj.6126/supp-10 Supplemental Information 10 Response curves of habitat suitability to environmental variables of *Magnolia biondii*. The x-axis is the value of environmental variable. The y-axis is the probability estimated by the Maxent which represents the habitat suitability.

10.7717/peerj.6126/supp-11 Supplemental Information 11 Response curves of habitat suitability to environmental variables of *Magnolia denudata*. The x-axis is the value of environmental variable. The y-axis is the probability estimated by the Maxent which represents the habitat suitability.

10.7717/peerj.6126/supp-12 Supplemental Information 12 Response curves of habitat suitability to environmental variables of *Magnolia sprengeri*. The x-axis is the value of environmental variable. The y-axis is the probability estimated by the Maxent which represents the habitat suitability.

10.7717/peerj.6126/supp-13 Supplemental Information 13 Receiver operating characteristic curves of *Magnolia biondii*, *Magnolia denudata* and *Magnolia sprengeri*.

10.7717/peerj.6126/supp-14 Supplemental Information 14 Spatial distribution of suitable habitats and national nature reserves.

10.7717/peerj.6126/supp-15 Supplemental Information 15 Spatial distribution of suitable habitats and land use/cover.

## Additional Information and Declarations

### Data Availability

The following information was supplied regarding data availability: Three Magnolia species distribution data: DOI 10.4121/uuid:c30a32cb-0b28-492d-a997-77dd002e14b4
