# Supplementary material for: Habitat differentiation and conservation gap of Magnolia biondii, M. denudata, and M. sprengeri in China
Source: PeerJ. 2019 Mar 12;6:e6126. doi: 10.7717/peerj.6126 (PMC6419747; doi:10.7717/peerj.6126)
Supplement: Supplemental Information 1 [file peerj-07-6126-s001.docx]

Table S1 Correlation coefficients between pairs of environmental variables

|  | altitude | aspect | slope | Annual mean temperature | Mean diurnal range | Isothermality | Temperature seasonality | Max temperature of warmest month | Min temperature of coldest month | Temperature annual range | Mean temperature of wettest quarter | Mean temperature of driest quarter | Mean temperature of warmest quarter | Mean temperature of coldest quarter | Annual precipitation | Precipitation of wettest month | Precipitation of driest month | Precipitation seasonality | Precipitation of wettest quarter | Precipitation of driest quarter | Precipitation of warmest quarter |
| --- | --- | --- | --- | --- | --- | --- | --- | --- | --- | --- | --- | --- | --- | --- | --- | --- | --- | --- | --- | --- | --- |
| aspect | 0.094 |  |  |  |  |  |  |  |  |  |  |  |  |  |  |  |  |  |  |  |  |
| slope | 0.138^*^ | 0.067 |  |  |  |  |  |  |  |  |  |  |  |  |  |  |  |  |  |  |  |
| Annual mean temperature | -0.814^**^ | -0.108 | -0.147^*^ |  |  |  |  |  |  |  |  |  |  |  |  |  |  |  |  |  |  |
| Mean diurnal range | 0.478^**^ | -0.203^**^ | 0.262^**^ | -0.349^**^ |  |  |  |  |  |  |  |  |  |  |  |  |  |  |  |  |  |
| Isothermality | 0.636^**^ | -0.171^*^ | 0.136^*^ | -0.260^**^ | 0.807^**^ |  |  |  |  |  |  |  |  |  |  |  |  |  |  |  |  |
| Temperature seasonality | -0.600^**^ | -0.041 | 0.064 | 0.113 | -0.233^**^ | -0.715^**^ |  |  |  |  |  |  |  |  |  |  |  |  |  |  |  |
| Max temperature of warmest month | -0.918^**^ | -0.115 | -0.082 | 0.884^**^ | -0.249^**^ | -0.411^**^ | 0.495^**^ |  |  |  |  |  |  |  |  |  |  |  |  |  |  |
| Min temperature of coldest month | -0.609^**^ | -0.011 | -0.230^**^ | 0.896^**^ | -0.404^**^ | -0.132^*^ | -0.215^**^ | 0.664^**^ |  |  |  |  |  |  |  |  |  |  |  |  |  |
| Temperature annual range | -0.300^**^ | -0.148^*^ | 0.206^**^ | -0.118 | 0.247^**^ | -0.303^**^ | 0.861^**^ | 0.308^**^ | -0.476^**^ |  |  |  |  |  |  |  |  |  |  |  |  |
| Mean temperature of wettest quarter | -0.574^**^ | -0.084 | -0.055 | 0.668^**^ | -0.137^*^ | -0.131 | 0.179^**^ | 0.613^**^ | 0.506^**^ | 0.089 |  |  |  |  |  |  |  |  |  |  |  |
| Mean temperature of driest quarter | -0.632^**^ | -0.055 | -0.115 | 0.897^**^ | -0.295^**^ | -0.100 | -0.151^*^ | 0.714^**^ | 0.939^**^ | -0.359^**^ | 0.406^**^ |  |  |  |  |  |  |  |  |  |  |
| Mean temperature of warmest quarter | -0.953^**^ | -0.103 | -0.117 | 0.914^**^ | -0.426^**^ | -0.523^**^ | 0.478^**^ | 0.963^**^ | 0.723^**^ | 0.208^**^ | 0.634^**^ | 0.741^**^ |  |  |  |  |  |  |  |  |  |
| Mean temperature of coldest quarter | -0.582^**^ | -0.058 | -0.161^*^ | 0.916^**^ | -0.292^**^ | -0.028 | -0.251^**^ | 0.671^**^ | 0.965^**^ | -0.449^**^ | 0.550^**^ | 0.945^**^ | 0.717^**^ |  |  |  |  |  |  |  |  |
| Annual precipitation | -0.510^**^ | 0.069 | -0.034 | 0.491^**^ | -0.431^**^ | -0.383^**^ | 0.092 | 0.464^**^ | 0.569^**^ | -0.184^**^ | -0.080 | 0.639^**^ | 0.484^**^ | 0.479^**^ |  |  |  |  |  |  |  |
| Precipitation of wettest month | -0.436^**^ | -0.018 | 0.030 | 0.537^**^ | -0.228^**^ | -0.148^*^ | -0.053 | 0.461^**^ | 0.583^**^ | -0.234^**^ | 0.066 | 0.658^**^ | 0.460^**^ | 0.553^**^ | 0.877^**^ |  |  |  |  |  |  |
| Precipitation of driest month | -0.582^**^ | 0.063 | -0.061 | 0.498^**^ | -0.496^**^ | -0.521^**^ | 0.267^**^ | 0.526^**^ | 0.493^**^ | -0.031 | -0.119 | 0.607^**^ | 0.556^**^ | 0.426^**^ | 0.887^**^ | 0.667^**^ |  |  |  |  |  |
| Precipitation seasonality | 0.493^**^ | -0.142^*^ | 0.083 | -0.154^*^ | 0.519^**^ | 0.715^**^ | -0.589^**^ | -0.346^**^ | -0.100 | -0.306^**^ | 0.159^*^ | -0.144^*^ | -0.375^**^ | 0.016 | -0.532^**^ | -0.118 | -0.725^**^ |  |  |  |  |
| Precipitation of wettest quarter | -0.418^**^ | -0.015 | 0.019 | 0.567^**^ | -0.256^**^ | -0.123 | -0.137^*^ | 0.447^**^ | 0.647^**^ | -0.327^**^ | 0.038 | 0.721^**^ | 0.456^**^ | 0.614^**^ | 0.908^**^ | 0.975^**^ | 0.717^**^ | -0.151^*^ |  |  |  |
| Precipitation of driest quarter | -0.590^**^ | 0.041 | -0.047 | 0.462^**^ | -0.469^**^ | -0.537^**^ | 0.332^**^ | 0.536^**^ | 0.440^**^ | 0.050 | -0.144^*^ | 0.562^**^ | 0.549^**^ | 0.366^**^ | 0.874^**^ | 0.649^**^ | 0.987^**^ | -0.758^**^ | 0.686^**^ |  |  |
| Precipitation of warmest quarter | -0.148^*^ | 0.021 | -0.029 | 0.334^**^ | -0.124 | 0.082 | -0.320^**^ | 0.172^*^ | 0.483^**^ | -0.419^**^ | 0.156^*^ | 0.441^**^ | 0.179^**^ | 0.438^**^ | 0.634^**^ | 0.818^**^ | 0.280^**^ | 0.182^**^ | 0.795^**^ | 0.244^**^ |  |
| Precipitation of coldest quarter | -0.570^**^ | 0.017 | -0.021 | 0.483^**^ | -0.414^**^ | -0.460^**^ | 0.265^**^ | 0.538^**^ | 0.484^**^ | 0.005 | -0.147^*^ | 0.613^**^ | 0.541^**^ | 0.411^**^ | 0.902^**^ | 0.694^**^ | 0.968^**^ | -0.713^**^ | 0.731^**^ | 0.979^**^ | 0.283^**^ |
