# Supplementary material for: Habitat differentiation and conservation gap of Magnolia biondii, M. denudata, and M. sprengeri in China
Source: PeerJ. 2019 Mar 12;6:e6126. doi: 10.7717/peerj.6126 (PMC6419747; doi:10.7717/peerj.6126)
Supplement: Supplemental Information 2 [file peerj-07-6126-s002.docx]

Table S2. The percent contribution and permutation importance of environmental variables

|  | *Magnolia biondii* | | *Magnolia denudata* | | *Magnolia sprengeri* | |
| --- | --- | --- | --- | --- | --- | --- |
| Variable | Percent contribution | Permutation importance | Percent contribution | Permutation importance | Percent contribution | Permutation importance |
| Altitude | 5.4 | 11.1 | 2.1 | 6.4 | 25.5 | 24.1 |
| Slope | 2.9 | 1.6 | 0.6 | 0 | 0.1 | 0.2 |
| Aspect | 1.4 | 1.1 | 0.9 | 1.2 | 1.2 | 1.4 |
| Mean diurnal range | 1 | 0 | 0.8 | 0.3 | 1.3 | 4.7 |
| Temperature seasonality | 8.1 | 14.2 | 8.5 | 8.4 | 13 | 23.8 |
| Min temperature of coldest month | 23.2 | 3.5 | 18.4 | 43.2 | 19.2 | 23.4 |
| Mean temperature of wettest quarter | 3.2 | 0 | 2.1 | 0.3 | 0.4 | 0.6 |
| Annual precipitation | 44.8 | 61.2 | 65.2 | 38 | 36.3 | 17.6 |
| Precipitation seasonality | 7.1 | 0.3 | 1.2 | 1.3 | 2.7 | 3 |
| Precipitation of warmest quarter | 2.9 | 7 | 0.2 | 0.9 | 0.3 | 1.1 |
