# Supplementary material for: Habitat differentiation and conservation gap of Magnolia biondii, M. denudata, and M. sprengeri in China
Source: PeerJ. 2019 Mar 12;6:e6126. doi: 10.7717/peerj.6126 (PMC6419747; doi:10.7717/peerj.6126)
Supplement: Supplemental Information 3 [file peerj-07-6126-s003.docx]

Table S3 The rank of topographic variables based on variable contribution

|  | *Magnolia biondii* | *Magnolia denudata* | *Magnolia sprengeri* |
| --- | --- | --- | --- |
| Percent contribution | Altitude,Slope,Aspect | Altitude, Aspect, Slope | Altitude, Aspect, Slope |
| Permutation importance | Altitude,Slope,Aspect | Altitude, Aspect, Slope | Altitude, Aspect, Slope |
| Training gain | Altitude,Slope,Aspect | Altitude, Aspect, Slope | Altitude,Slope,Aspect |
| AUC | Altitude,Slope,Aspect | Altitude, Aspect, Slope | altitude, aspect, slope |
| Test gain | Altitude,Slope,Aspect | Altitude, Aspect, Slope | Altitude,Slope,Aspect |
