# Supplementary material for: Habitat differentiation and conservation gap of Magnolia biondii, M. denudata, and M. sprengeri in China
Source: PeerJ. 2019 Mar 12;6:e6126. doi: 10.7717/peerj.6126 (PMC6419747; doi:10.7717/peerj.6126)
Supplement: Supplemental Information 4 [file peerj-07-6126-s004.docx]

Table S4 The rank of temperature variables based on variable contribution

|  | *Magnolia biondii* | *Magnolia denudata* | *Magnolia sprengeri* |
| --- | --- | --- | --- |
| Percent contribution | Min temperature of coldest month  Temperature seasonality  Mean temperature of wettest quarter  Mean diurnal range | Min temperature of coldest month  Temperature seasonality  Mean temperature of wettest quarter  Mean diurnal range | Min temperature of coldest month  Temperature seasonality  Mean diurnal range  Mean temperature of wettest quarter |
| Permutation importance | Temperature seasonality  Min temperature of coldest month  Mean temperature of wettest quarter  Mean diurnal range | Min temperature of coldest month  Temperature seasonality  Mean temperature of wettest quarter  Mean diurnal range | Min temperature of coldest month  Temperature seasonality  Mean diurnal range  Mean temperature of wettest quarter |
| Training gain | Min temperature of coldest month  Mean diurnal range  Temperature seasonality  Mean temperature of wettest quarter | Min temperature of coldest month  Mean diurnal range  Temperature seasonality  Mean temperature of wettest quarter | Min temperature of coldest month  Temperature seasonality  Mean diurnal range  Mean temperature of wettest quarter |
| AUC | Min temperature of coldest month  Temperature seasonality  Mean diurnal range  Mean temperature of wettest quarter | Min temperature of coldest month  Mean diurnal range  Temperature seasonality  Mean temperature of wettest quarter | Min temperature of coldest month  Temperature seasonality  Mean diurnal range  Mean temperature of wettest quarter |
| Test gain | Min temperature of coldest month  Mean diurnal range  Temperature seasonality  Mean temperature of wettest quarter | Min temperature of coldest month  Mean diurnal range  Temperature seasonality  Mean temperature of wettest quarter | Min temperature of coldest month  Temperature seasonality  Mean diurnal range  Mean temperature of wettest quarter |
