# Supplementary material for: Habitat differentiation and conservation gap of Magnolia biondii, M. denudata, and M. sprengeri in China
Source: PeerJ. 2019 Mar 12;6:e6126. doi: 10.7717/peerj.6126 (PMC6419747; doi:10.7717/peerj.6126)
Supplement: Supplemental Information 5 [file peerj-07-6126-s005.docx]

Table S5 The rank of precipitation variables based on variable contribution

|  | *Magnolia biondii* | *Magnolia denudata* | *Magnolia sprengeri* |
| --- | --- | --- | --- |
| Percent contribution | Annual precipitation  Precipitation seasonality  Precipitation of warmest quarter | Annual precipitation  Precipitation seasonality  Precipitation of warmest quarter | Annual precipitation  Precipitation seasonality  Precipitation of warmest quarter |
| Permutation importance | Annual precipitation  Precipitation of warmest quarter  Precipitation seasonality | Annual precipitation  Precipitation seasonality  Precipitation of warmest quarter | Annual precipitation  Precipitation seasonality  Precipitation of warmest quarter |
| Training gain | Annual precipitation  Precipitation seasonality  Precipitation of warmest quarter | Annual precipitation  Precipitation of warmest quarter  Precipitation seasonality | Annual precipitation  Precipitation seasonality  Precipitation of warmest quarter |
| AUC | Annual precipitation  Precipitation seasonality  Precipitation of warmest quarter | Annual precipitation  Precipitation of warmest quarter  Precipitation seasonality | Precipitation seasonality  Annual precipitation  Precipitation of warmest quarter |
| Test gain | Annual precipitation  Precipitation seasonality  Precipitation of warmest quarter | Annual precipitation  Precipitation of warmest quarter  Precipitation seasonality | Annual precipitation  Precipitation seasonality  Precipitation of warmest quarter |
