# Supplementary material for: Habitat differentiation and conservation gap of Magnolia biondii, M. denudata, and M. sprengeri in China
Source: PeerJ. 2019 Mar 12;6:e6126. doi: 10.7717/peerj.6126 (PMC6419747; doi:10.7717/peerj.6126)
Supplement: Supplemental Information 6 [file peerj-07-6126-s006.docx]

Table S6 Thresholds estiamted by Maxent for the fitted model of *Magnolia biondii*

| Cumulative threshold | Cloglog threshold | Description | Fractional predicted area | Training omission rate | Test omission rate |
| --- | --- | --- | --- | --- | --- |
| 1.000 | 0.034 | Fixed cumulative value 1 | 0.305 | 0.017 | 0.015 |
| 5.000 | 0.107 | Fixed cumulative value 5 | 0.205 | 0.034 | 0.031 |
| 10.000 | 0.208 | Fixed cumulative value 10 | 0.153 | 0.080 | 0.071 |
| 0.647 | 0.022 | Minimum training presence | 0.325 | 0.000 | 0.000 |
| 14.558 | 0.297 | 10 percentile training presence | 0.127 | 0.097 | 0.087 |
| 16.433 | 0.329 | Equal training sensitivity and specificity | 0.119 | 0.119 | 0.107 |
| 14.073 | 0.286 | Maximum training sensitivity plus specificity | 0.129 | 0.091 | 0.082 |
| 18.393 | 0.360 | Equal test sensitivity and specificity | 0.111 | 0.125 | 0.112 |
| 14.073 | 0.286 | Maximum test sensitivity plus specificity | 0.129 | 0.091 | 0.082 |
| 0.647 | 0.022 | Balance training omission, predicted area and threshold value | 0.325 | 0.000 | 0.000 |
| 8.171 | 0.169 | Equate entropy of thresholded and original distributions | 0.168 | 0.074 | 0.066 |

“Fractional predicted area” is the fraction of the total study area predicted present. “Test omission rate” is the proportion of the test localities falling outside the prediction. “Training omission rate” is the proportion of the train localities falling outside the prediction.
