# Supplementary figures and images for: Habitat differentiation and conservation gap of Magnolia biondii, M. denudata, and M. sprengeri in China
Source: PeerJ. 2019 Mar 12;6:e6126. doi: 10.7717/peerj.6126 (PMC6419747; doi:10.7717/peerj.6126)

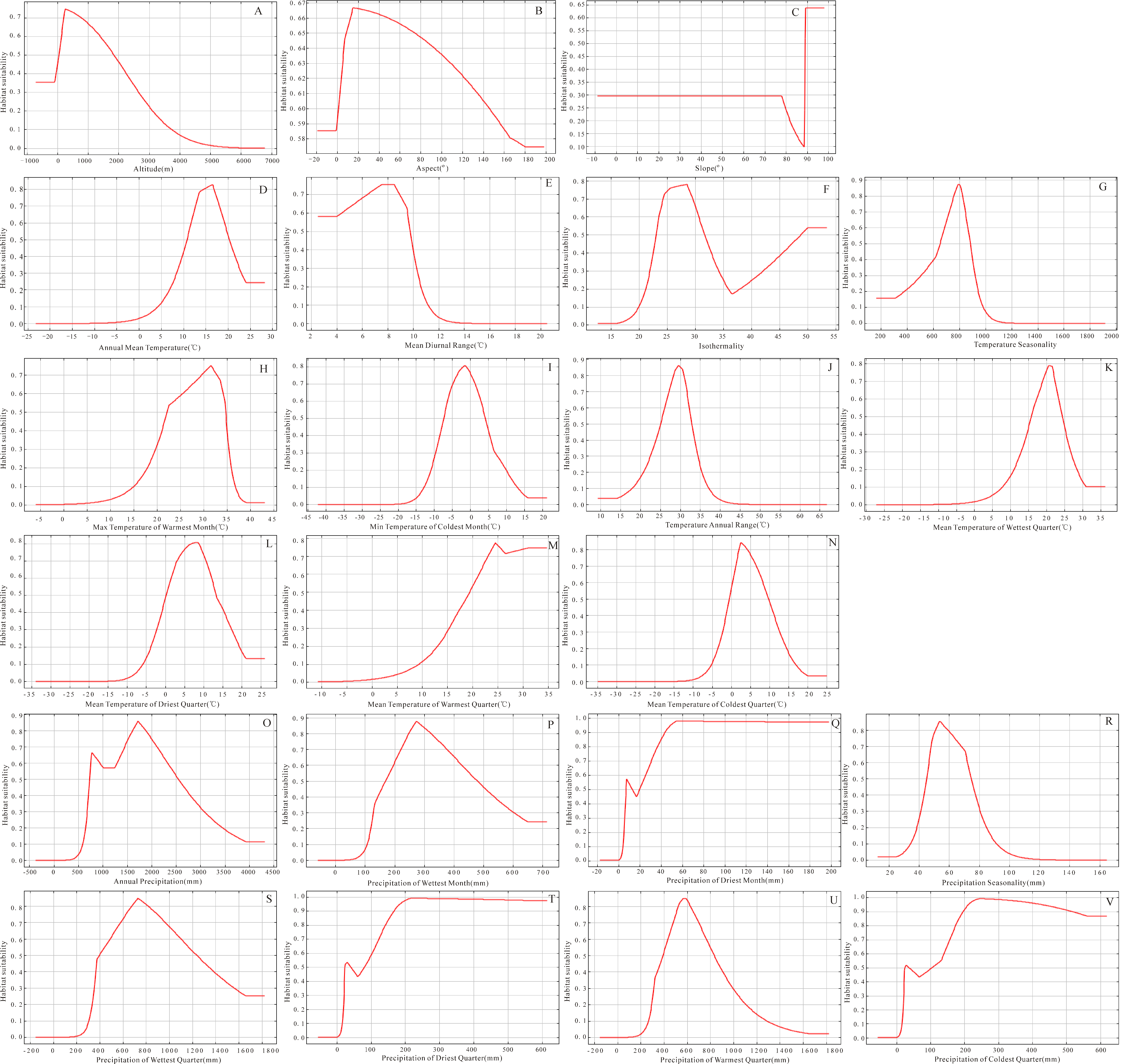

Supplement: Supplemental Information 10 — The x-axis is the value of environmental variable. The y-axis is the probability estimated by the Maxent which represents the habitat suitability. [file peerj-07-6126-s010.png]

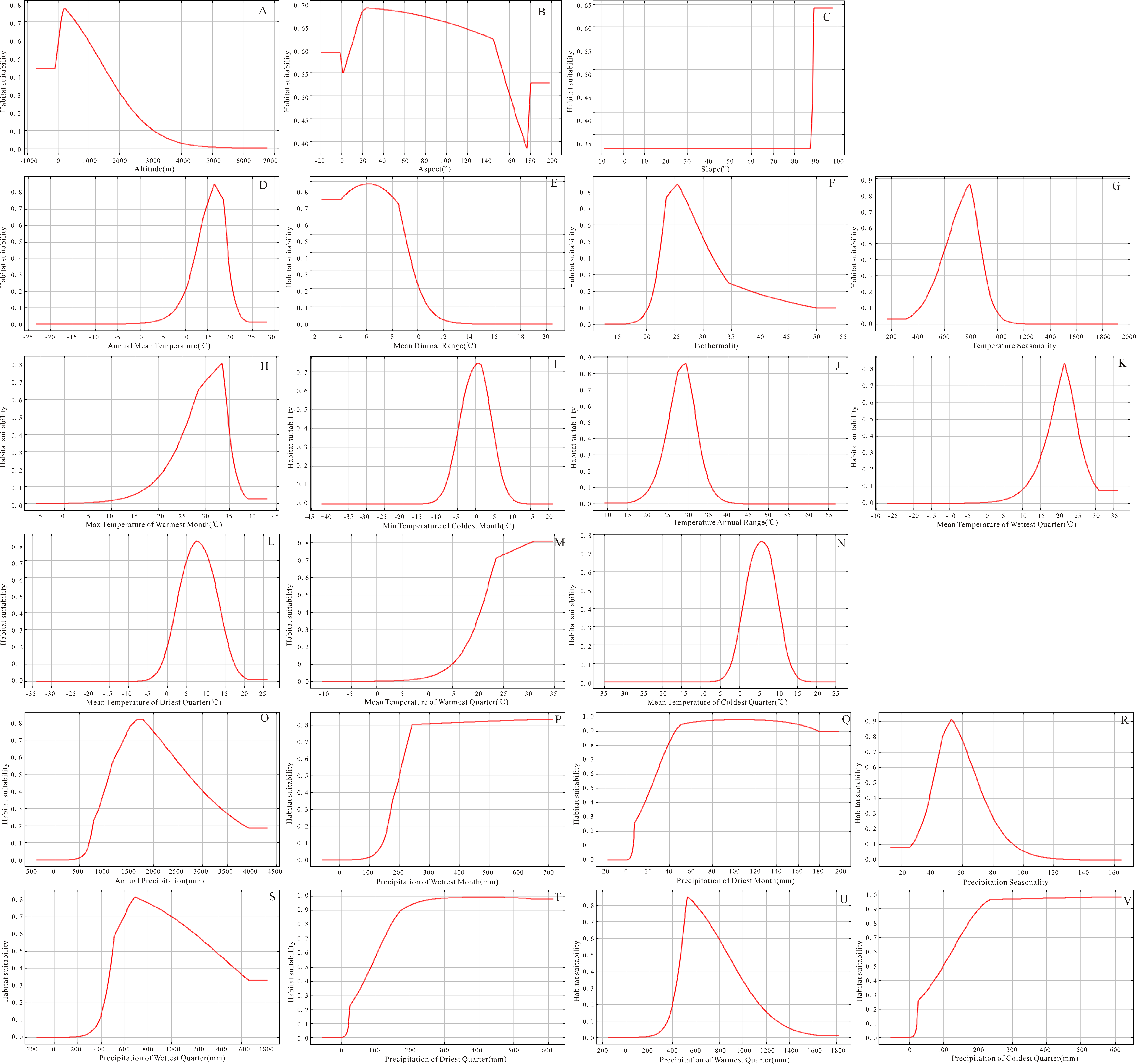

Supplement: Supplemental Information 11 — The x-axis is the value of environmental variable. The y-axis is the probability estimated by the Maxent which represents the habitat suitability. [file peerj-07-6126-s011.png]

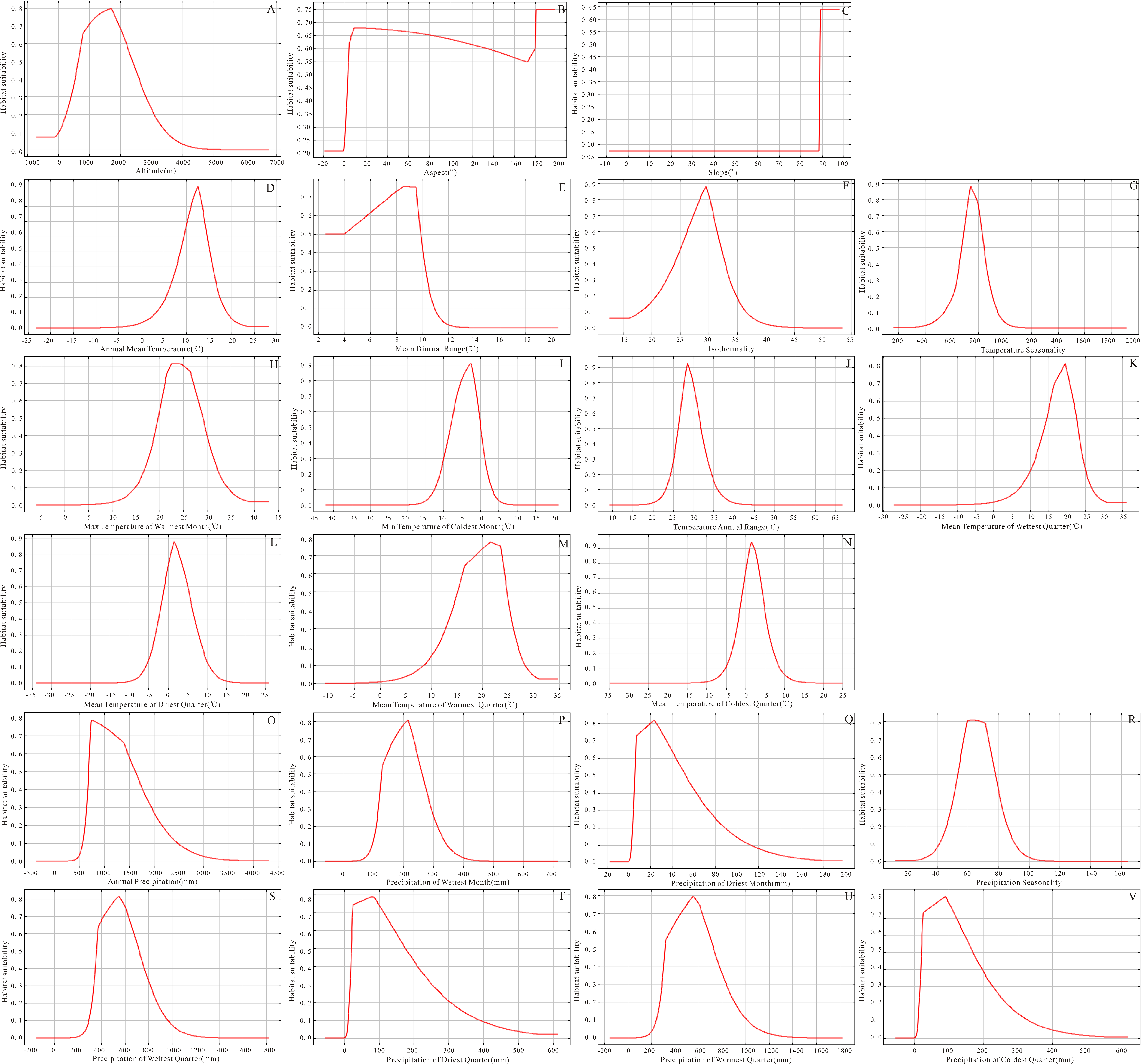

Supplement: Supplemental Information 12 — The x-axis is the value of environmental variable. The y-axis is the probability estimated by the Maxent which represents the habitat suitability. [file peerj-07-6126-s012.png]

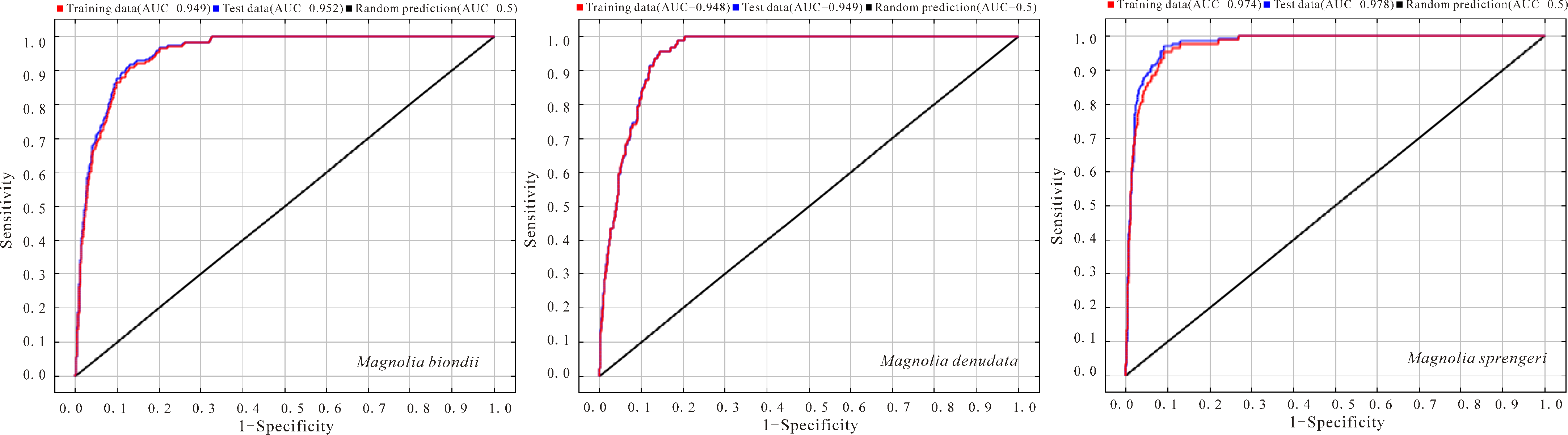

Supplement: Supplemental Information 13 [file peerj-07-6126-s013.png]

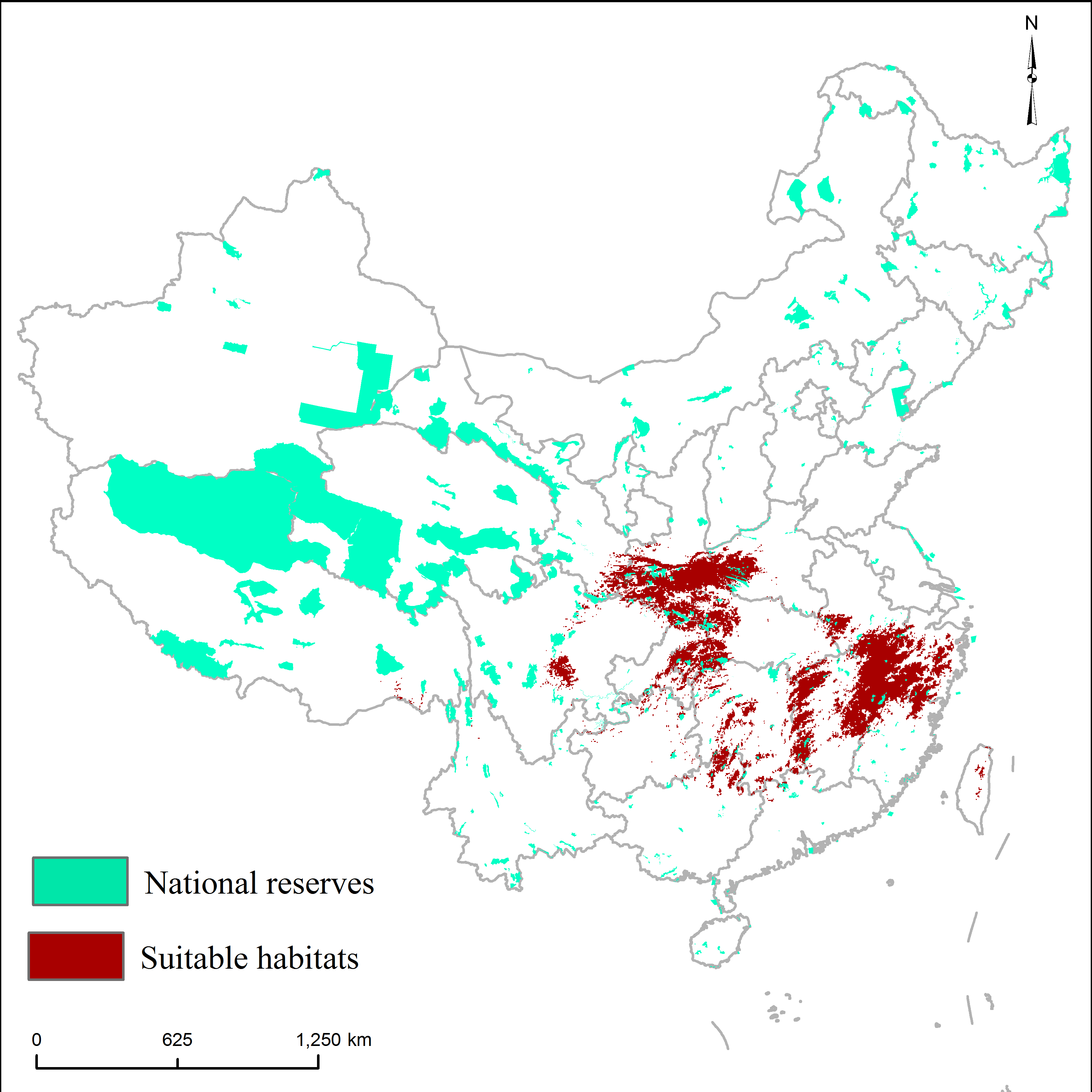

Supplement: Supplemental Information 14 [file peerj-07-6126-s014.png]

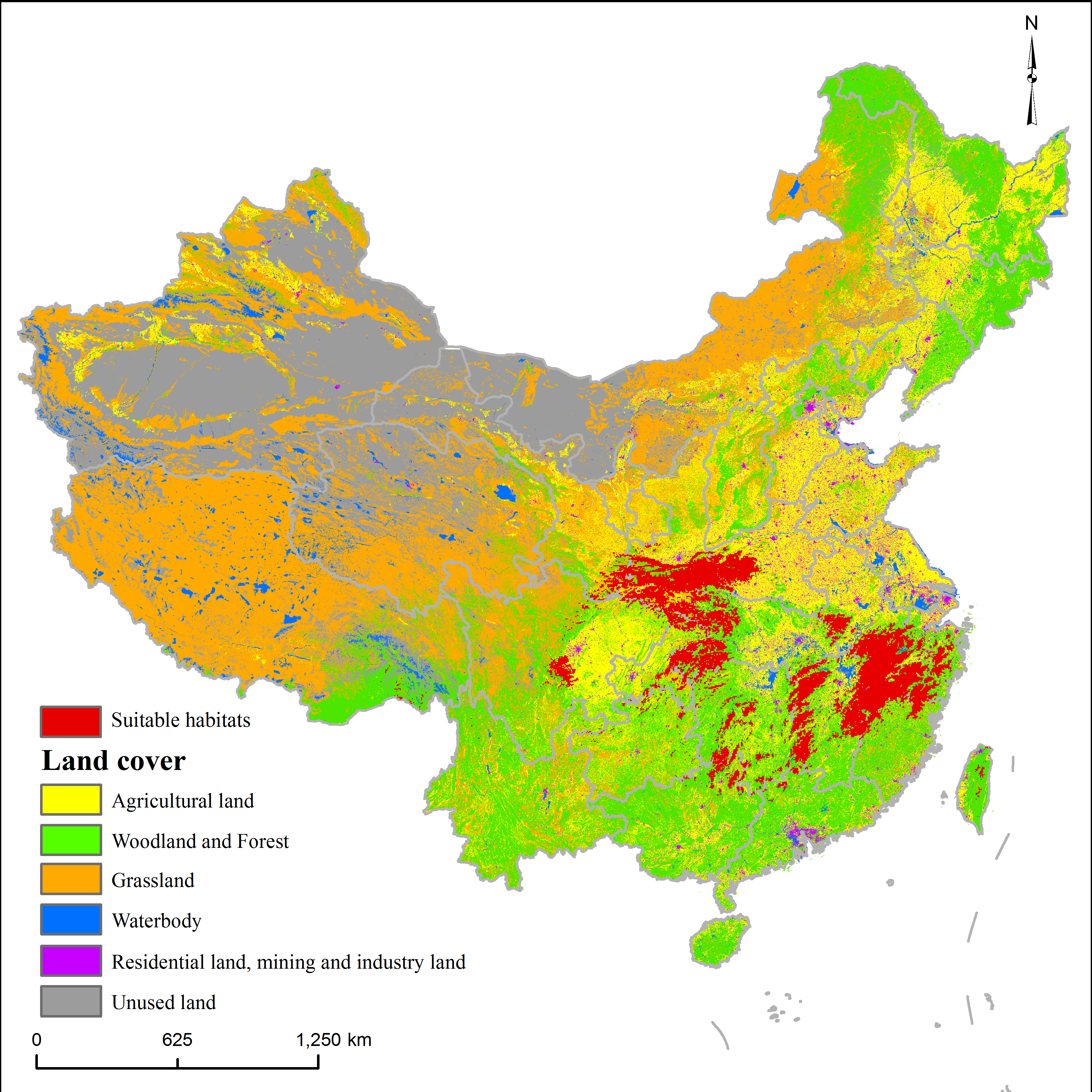

Supplement: Supplemental Information 15 [file peerj-07-6126-s015.png]
